# Supplementary material for: Identification of photoperiod-induced specific miRNAs in the adrenal glands of Sunite sheep (Ovis aries)
Source: Front Vet Sci. 2022 Jul 22;9:888207. doi: 10.3389/fvets.2022.888207 (PMC9354845; doi:10.3389/fvets.2022.888207)
Supplement: Supplementary file 1 [file Data_Sheet_1.ZIP › Supplementary file/miRDeep2 file.zip!/miRDeep2 file/Know_Structure/LP42A3/oar-let-7c.pdf]

miRBase precursor : oar-let-7c
Total read count : 120280
oar-let-7c read count : 120209
remaining reads : 71

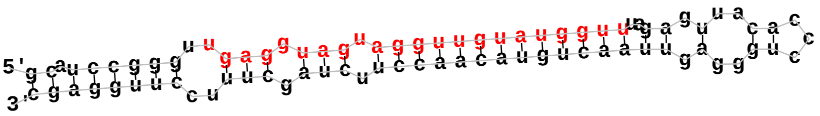

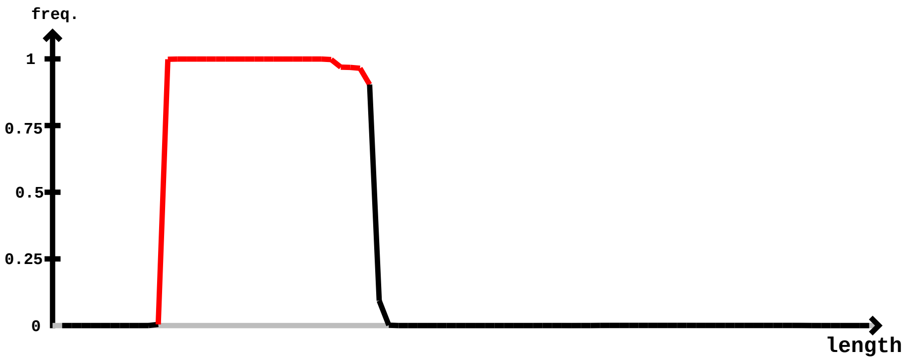

oar-let-7c

```
5'- gcauccgggu ugagguaguagguuguaugguu uagaguuacacccugggaguuaacuguacaaccuucuagcuuuccuuggagc   -3'   exp
    ((.((((((..(((.(((.((((((((((((((..((.(..((...))..).))))))))))))))).))).)))..))))))))   reads   mm   sample
    .......gguugagguaguagguuguaugguu.....................................................   1       0    seq
    ........guugagguaguagguuguaug........................................................   1       0    seq
    ........guugagguaguagguuguaugg.......................................................   2       0    seq
    ........guugagguaguagguuguauggu......................................................   4       0    seq
    ........guugagguaguagguuguaugguu.....................................................   3       0    seq
    .........uugagguaguagguuguau.........................................................   2       0    seq
    .........uugagguaguagguuguaugg.......................................................   9       0    seq
    .........uugagguaguagguuguauggu......................................................   137     0    seq
    .........uugagguaguagguuguaugguu.....................................................   338     0    seq
    .........uugagguaguagguuguauAguu.....................................................   9       1    seq
    .........uugagguaguagguuguaugguuu....................................................   24      0    seq
    .........uugagguaguagguuguauAguuu....................................................   1       1    seq
    ..........ugagguaguagguugua..........................................................   216     0    seq
    ..........ugagguagGagguugua..........................................................   24      1    seq
    ..........ugagguaguaAguugua..........................................................   5       1    seq
    ..........ugaAguaguagguugua..........................................................   1       1    seq
    ..........ugagguagGagguuguau.........................................................   362     1    seq
    ..........ugaAguaguagguuguau.........................................................   28      1    seq
    ..........ugagguaguagguuUuau.........................................................   8       1    seq
    ..........ugagguaguaAguuguau.........................................................   22      1    seq
    ..........ugagguUguagguuguau.........................................................   3       1    seq
    ..........ugagguaguagguuguau.........................................................   2991    0    seq
    ..........ugagguaguagguuguaug........................................................   109     0    seq
    ..........ugagguaguagguuguaugg.......................................................   376     0    seq
    ..........ugagguaguagguuguauggu......................................................   7196    0    seq
    ..........ugagguaguagguuguaugguu.....................................................   97035   0    seq
    ..........ugagguaguagguuguaugguuu....................................................   11023   0    seq
    ..........ugagguaguagguuguauAguuua...................................................   100     1    seq
    ..........ugagguaguagguuguaugguuua...................................................   62      0    seq
    ..........ugagguaguagguuguaugguuuag..................................................   2       0    seq
    ..........ugagguaguagguuguaugguuuaga.................................................   1       0    seq
    ..........ugagguaguagguuguaugguuuagaguuac............................................   1       0    seq
    ............gagguaguagguuguau........................................................   2       0    seq
    ............gagguagGagguuguau........................................................   1       1    seq
    ............gagguaguagguuguaugg......................................................   1       0    seq
```

oar-let-7c

```
gcauccggguugagguaguagguuguaugguuuagaguuacacccugggaguuaacuguacaaccuucuagcuuuccuuggagc
...........gagguaguagguuguauggu.....................................................	6	0	seq
...........gagguaguagguuguaugguu....................................................	91	0	seq
...........gagguaguagguuguaugguuu...................................................	10	0	seq
...........gagguaguagguuguaugguuua..................................................	1	0	seq
............agguaguagguuguaugguu....................................................	3	0	seq
................................uagaguuacacccugggagu................................	1	0	seq
................................uagaguuacacccugggaguu...............................	1	0	seq
.........................................acccugggaguuaacuguacaacc...................	1	0	seq
..........................................cccugggaguuaacuguacaacc...................	1	0	seq
...............................................ggaguuaacuguacaaccuucu...............	1	0	seq
.................................................aguuaacuguacaaccuucuagc............	1	0	seq
.................................................aguuaacuguacaaccuucuagcu...........	1	0	seq
.....................................................aacuguacaaccuucuagcuu..........	1	0	seq
.....................................................aacuguacaaccuucuagcuuu.........	2	0	seq
.....................................................aacuguacaaccuucuagcuuuc........	1	0	seq
.......................................................cuguacaaccuucuagcuuu.........	1	0	seq
.......................................................cuguacaaccuucuagcuuuc........	8	0	seq
.......................................................cuguacaaccuucuagcuuucc.......	42	0	seq
.......................................................cuguacaaccuucuagcuuuccu......	5	0	seq
........................................................uguacaaccuucuagcuuuccu......	2	0	seq
```
